# Therapeutic potential of lactoferrin-coated iron oxide nanospheres for targeted hyperthermia in gastric cancer

**DOI:** 10.1038/s41598-023-43725-3

**Published:** 2023-10-19

**Authors:** Komal Attri, Bhupendra Chudasama, Roop L. Mahajan, Diptiman Choudhury

**Affiliations:** 1 School of Chemistry and Biochemistry, Thapar Institute of Engineering and Technology, Patiala, Punjab 147004 India; 2 School of Physics and Material Sciences, Thapar Institute of Engineering and Technology, Patiala, Punjab 147004 India; 3 Department of Mechanical Engineering, Department of Materials Science and Engineering, Virginia Tech, Blacksburg, VA 24061 USA; 4 TIET-VT Centre of Excellence for Emerging Materials, Thapar Institute of Engineering and Technology, Patiala, Punjab 147004 India

**Keywords:** Biochemistry, Biological techniques, Cancer, Drug discovery, Diseases, Health care, Medical research, Oncology

## Abstract

Lactoferrin (LF) is a non-heme iron-binding glycoprotein involved in the transport of iron in blood plasma. In addition, it has many biological functions, including antibacterial, antiviral, antimicrobial, antiparasitic, and, importantly, antitumor properties. In this study, we have investigated the potential of employing lactoferrin-iron oxide nanoparticles (LF-IONPs) as a treatment modality for gastric cancer. The study confirms the formation of LF-IONPs with a spherical shape and an average size of 5 ± 2 nm, embedded within the protein matrix. FTIR and Raman analysis revealed that the Fe–O bond stabilized the protein particle interactions. Further, we conducted hyperthermia studies to ascertain whether the proposed composite can generate a sufficient rise in temperature at a low frequency. The results confirmed that we can achieve a temperature rise of about 7 °C at 242.4 kHz, which can be further harnessed for gastric cancer treatment. The particles were further tested for their anti-cancer activity on AGS cells, with and without hyperthermia. Results indicate that LF-IONPs (10 µg/ml) significantly enhance cytotoxicity, resulting in the demise of 67.75 ± 5.2% of cells post hyperthermia, while also exhibiting an inhibitory effect on cell migration compared to control cells, with the most inhibition observed after 36 h of treatment. These findings suggest the potential of LF-IONPs in targeted hyperthermia treatment of gastric cancer.

## Introduction

Despite the development of various strategies, therapies, and drugs for the diagnosis and treatment of cancer, it remains a major cause of death worldwide. The available cancer treatments, such as chemotherapy drugs, have inherent limitations as they destroy both malignant and healthy cells, resulting in the destruction of metabolically active cells in the body, suppression of the immune system, systemic toxicity, and an increased risk of secondary infections in cancer patients^1,2^. As drug resistance becomes increasingly prevalent, there is a growing demand for natural agents that can eradicate primary tumors and reduce the risk of recurrence^3^. One such natural agent is lactoferrin (LF), a protein belonging to the transferrin family, with a molecular weight of 78 kDa and consisting of approximately 690 amino acid residues^4^. Although the primary function of LF in humans is to transport iron in blood plasma, it also possesses several biological functions, including antibacterial, antiviral, antimicrobial, antiparasitic, and, notably, antitumor activities^5,6^. The highest concentration of LF is found in human milk, followed by cow's milk^7^. Lactoferrin is composed of a single polypeptide chain that encompasses two lobes (N and C) joined by an α-helical residue. This structural arrangement provides flexibility^3,8^. These two lobes consist of α-helices and β-sheets and can bind both Fe^2+^ or Fe^3+^ ions in synergy with carbonate ions (CO_3_^2−^)^8^. The LF protein exists in two forms: Apo-lactoferrin (iron-free form) and holo-lactoferrin (iron-containing form)^4^. In its natural state, LF is partially saturated with iron. However, it can also become fully saturated with iron from an external source^9,10^. Lactoferrin (LF) has been extensively studied for its potential as a natural agent in combating various types of cancer, including gastric cancer, and has been shown to be a highly efficient bio-drug in anti-cancer research. Several in-vitro and in-vivo studies have reported that LF can inhibit the growth of tumor cells through diverse mechanisms, including apoptosis, cell cycle arrest, cell membrane disruption, immunoreaction, decreased cell migration, and cytoskeleton damage^11–15^. It is a very stable protein that can retain its effectiveness even after passing through the gastrointestinal tract^16^. Studies have shown that oral administration of LF results in a decreased occurrence of tongue (2% bLF), esophageal (0.2% bLF), and lung (0.02% bLF) carcinogenesis in rats, with no discernible impact on the weight of the body organs, indicative of its non-toxicnature^17,18^. Additionally, LF has been shown to induce cytotoxicity and decrease cell proliferation in MCF-7 and MDA-MB-231 human breast cancer cell lines^19,20^.

Lactoferrin is stored in the secondary cytoplasmic granules of neutrophils and is found in high concentrations at the sites of inflammation, playing a vital role in the inflammatory response mechanism^21^. Moreover, the kidneys synthesize LF, contributing to the immune defence system by sequestering free iron from urine and making it available for metabolic functions^22^. At the cellular level, LF modulates the maturation, migration, differentiation, activation, proliferation, and functions of immune cells by using two signaling pathways^23^. Accumulated LF in neutrophils at injury sites promotes cell–cell interaction, activates phagocytosis by polymorphonuclear leukocytes and macrophages, decreases the number of pro-inflammatory cytokines, increases natural killer cell activity, and activates lymphocytes^24,25^. Furthermore, LF also plays a valuable role in distinguishing normal cells from tumor cells. Tumor cells typically possess a highly negative charge compared to normal cells, rendering them more susceptible to cationic proteins like LF, while sparing the normal cells^26,27^. Studies have shown that many cancer cells possess elevated levels of proteoglycan, glycosaminoglycan, and sialic acid, which interact with LF protein and exert a cytotoxic effect on cancer cells. This mechanism explains LF's ability to selectively target and exert high cytotoxicity selectivity toward cancer cells while posing no harm to healthy cells^28–30^. Magnetic nanoparticles, when properly synthesized and made biocompatible, play a crucial role in various biomedical applications, ranging from imaging to therapy^31^. To serve as efficient carriers for drug delivery, they should be small in size, have a large surface-to-volume ratio, and be properly functionalized for site-specific targeting^32^. Among the various types of magnetic nanoparticles, iron oxide nanoparticles find common use due to their magnetic properties and biocompatibility across a variety of biomedical applications, including drug and gene delivery, biosensors, magnetic particle imaging (MPI), and hyperthermia (HM) treatment^33–35^. Superparamagnetic iron oxide nanoparticles can be engineered to respond specifically and efficiently within the tumor microenvironment^36–38^. These particles can be magnetized by an external magnetic field but exhibit no residual magnetic interactions after the removal of the field, indicating good dispersion and excellent targeting capacity^39^. This characteristic makes them highly advantageous for magnetic hyperthermia due to their ability to disperse within localized minor regions, creating a difference in temperature profiles between normal and tumor cells^40^.

Various particles have been synthesized to harness their anti-cancer potential against different cancers by using hyperthermia treatments. Mohamadkazem et al. synthesized iron oxide-gold nanocomplexes and used an external field to physically navigate these magnetic nanoparticles to the target melanoma cells, effectively killing them through electron beam therapy^41^. Kamalabadi et al. synthesized folate-functionalized gold-coated magnetic nanoparticles for treating HPV-positive oropharyngeal cancer by enhancing uptake and cell death through the application of an external field^42^. Further, hyperthermia has been found to enhance drug release from formulations and developing potent theranostic agents for anti-cancer activity against colorectal cancer^43^.

Based on the above, we hypothesized that the targeted delivery of lactoferrin conjugated with iron oxide nanoparticles (LF-IONPs) to gastric tissue, coupled with hyperthermia, will offer enhanced efficacy in the treatment of gastric cancer.

## Materials and methods

### Materials

Ferric chloride (FeCl_3_), ferrous sulfate (FeSO_4_), ammonium hydroxide (NH_4_OH), polyethylene glycol (PEG 400), and deionized water of analytical grade were purchased from LobaChemie, India. Lactoferrin, ammonium hydroxide, ethylene dichloride (EDC), N-hydroxysuccinimide (NHS), HAMs cell culture media, fetal bovine serum (FBS), and penicillin–streptomycin were purchased from HiMedia, India.

### Synthesis of iron oxide nanoparticles followed by conjugation with Lactoferrin

To synthesize iron oxide nanoparticles, FeCl_3_·6H_2_O and FeSO_4_·7H_2_O were dissolved in 50 ml of deionized water and heated to 90 °C, followed by the addition of 3 ml PEG400. Subsequently, a separate solution containing 10 ml of 25% ammonium hydroxide in 50 ml of water was rapidly added to the iron solutions while stirring, followed by a stirring period of 30 min. The resulting mixture turned black and was then cooled to room temperature, centrifuged at 4000 rpm for 10 min, and washed five times with water to remove any undissolved impurities.

To conjugate the nanoparticles with LF protein, a solution was prepared by mixing 250 µl of 10 mg/ml EDC, 250 µl of 10 mg/ml NHS, and 7 µl of 1 M NaOH. The synthesized iron oxide nanoparticles were added (0.5 mg) and sonicated for 15 min to activate the carboxyl group on the surface. Next, 250 µl of 3 mg/ml LF was added and left overnight at room temperature. The resultant solution was purified by centrifugation at 12,000*g* for 1 h and redispersed in phosphate buffer saline (PBS) three times to obtain a pure nano-formulation.

### Study of particle size, morphology, and elemental analysis

The Malvern DLS-Zeta size analyzer was used to determine the hydrodynamic size and surface charge of the nanoparticles through DLS (dynamic light scattering) and zeta studies, respectively. Scanning Electron Microscopy (SEM JEOL, JSM-6300) was used for visualizing small topographic details on the surface, while high-resolution transmission electron microscopy (HRTEM) (Talos F200S G2, Thermo Scientific) was employed to identify the size, shape, and interface structure of LF-IONPs. Prior to analysis, the nanospheres were centrifuged at 240 rpm for 15 min and washed to eliminate any unbound metal salt or impurities. The resulting pellet was analyzed using an energy dispersive X-ray spectrometer (EDS) (Bruker QUANTAX 200) to determine the percentage of present elements.

### Study of LF-IONPs interaction using spectroscopy

Fourier transform infrared spectroscopy (FTIR) is a technique used to determine the chemical bonds in a molecule by producing an infrared absorption spectrum through discrete vibrational energy of functional groups. It was employed to identify the interactions between LF protein and iron oxide nanoparticles. The FTIR studies were performed using an Agilent Cary 600 series Spectrophotometer, and the samples were prepared using the potassium bromide (KBr) method and scanned from 400 to 4000 cm^−1^.

Surface enhanced Raman scattering (SERS) Spectra were also utilized to monitor the structural changes in the protein after conjugation with iron oxide particles. Thin films of LF, IONPs and LF-IONPs were prepared 10 min prior to measurement on glass slides. The samples were then subjected to scanning from 500 to 1800 cm^−1^ using the LabRam Hr Evolution Horiba instrument, equipped with a detector and microscope to record the Raman spectra for the samples.

### In silico studies

To identify the binding site residues of different transition metal ions, the MIB (metal ion binding) online docking tool can be used^44^. It is done by using the fragment transformation method. The process begins with the selection of a protein of interest, which was extracted from the protein data bank (PDB). Then, the query protein is compared with each metal ion in the database to find the metal binding residues, and a score is assigned to each binding residue.

The fragment transformation method is used to align the query protein S of length m and metal-binding template T of n residues. These chains are then aligned in such a manner that the metal ion binding protein template can be converted into the query protein structure^44^. Certain parameters are being taken into consideration to get these protein structures. Firstly, the metal ion template must contain residues bound with transition metals, including Ni^2+^, Cu^2+^, Mg^2+^, Ca^2+^, Co^2+^, Zn^2+^, Fe^2+^, and Fe^3+^ metal ions. Secondly, the length of the polypeptide chain in protein structures must be 50 residues in order to be included for docking purposes^45^. The residues of the query template and metal ion binding triplets can be represented by using the notation N–Ca–C, denoting the backbone atoms as (xN, xCa, xC) and (yN, yCa, yC), where x and y are PDB coordinates. The query protein S and the template T can be written as (s1, s2.sm) and (s1, s2.sm) in terms of triplets. The third parameter is that there should be at least two metal ion binding residues^44^. The fourth important parameter is that the residue's binding score should be more than a specified threshold value in order for it to be considered as a residue binding to a particular transition metal ion. The binding score, denoted by Ci, is assigned to all residues and of the target protein based on the sequence and structural conservation of the protein using the root mean square deviation of C-alpha carbons from structural local alignment as well as BLOSUM62 substitution matrix^46^. Hence, the binding site of metal ions was determined using the bioinformatics tool^47^. In this way, the MIB tool helps determine the binding site of metal ions on a particular protein chain. For example, in case of LF protein (PDB ID: 4EWW) extracted from the PDB (Protein Data Bank) database, a single chain denoted by A, Fe^2+^ and Fe^3+^ metal ions were docked with LF and metal ion-binding templates were compared with the target protein.

### XRD analysis

X-ray diffraction pattern gives us deep knowledge about the chemical composition, crystallographic structure, and physical properties of a material. For XRD analysis, the sample was first dried and then subjected to the Cu K α radiation (λ = 1.54 Å) to obtain the diffraction patterns of the material. The diffraction pattern was collected in the 2Ɵ scan range of 10°–90°. Then, the average crystallite size of IONPs and LF-IONPs was determined by employing the highest intense peak by using the Scherrer formula.

1 $$d=\frac{K\lambda }{\beta cos\theta}$$

where K = 0.9, a Scherrer constant, λ = 1.54 Å is the wavelength of the X-rays, β is the broadening of the highest intense peak, and d is the crystallite size of the synthesized nanoparticles, which includes IONPs (Iron oxide nanoparticles) and LF-IONPs (Lactoferrin-Iron oxide nanospheres).

### Hyperthermia study to check the heating capacity

Hyperthermia is a type of heat treatment that involves raising the temperature of tumor-loaded tissue to between 42 and 45 °C^48,49^. This type of treatment is used to kill cancer cells with little or no harm to normal cells. In order to perform hyperthermia analysis of synthesized nanospheres, we used the NanoTherics Magnetherm magnetic hyperthermia instrument. It consists of an optical fiber temperature probe. This involves analyzing the nanospheres under a 10 mT magnetic field and at various frequencies (including 161.9 kHz, 242.4 kHz, 411.1 kHz, 580.2 kHz, and 935.3 kHz) to determine the lowest frequency at which the particles generate a safe and effective temperature rise for treatment purposes.

### VSM study to check the magnetic properties

A Vibrating sample magnetometer (VSM, Lake Shore 7404) was used to analyze the magnetic properties of the iron oxide nanoparticles. The sample was subjected to a + /− 10 kilo-oersted magnetic field at room temperature. From the obtained hysteresis loop (M-H), saturation magnetization of the powdered form of the sample was recorded. The hysteresis loop explains the relationship between magnetic flux density and the magnetizing field strength and is a characteristic of ferromagnetic materials. By analyzing the hysteresis loop, important magnetic properties such as saturation magnetization (Ms), coercivity (Hc), and remanence (M_r_) can be obtained^50^_._

### Drug loading and release kinetics

For studying the drug loading efficiency of the synthesized LF-IONPs, 1 ml of the sample was centrifuged at 10,000 rpm for 15 min. The concentration of the protein LF was determined both for the supernatant (unbound protein) as well as a pellet (bound protein) by using a Bradford reagent. Thereafter, the release kinetics was performed by placing particles in a dialysis membrane with a drug concentration of 3 mg/ml at a temperature of 37 °C. The entire process of release kinetics was performed at two different pH; pH 7.4 (physiological pH) and pH 3 (gastric pH). For performing the studies, the dialysis membrane was suspended in a beaker with distilled water of pH 7.4 (physiological pH) and a volume of 50 ml. The 1 ml sample was collected from the beaker at different time intervals, followed by the addition of a similar volume of distilled water to the beaker to maintain the total volume constant. Similar process was followed for studying the release kinetics at the gastric pH (pH of water = 3) to monitor the efficient release of drug at the target site. Thereafter, the rate of release of bound LF from the pellet was observed at different time intervals for about 40 h by taking absorption values at 595 nm. Then, these absorption values were plotted to infer the trend of the release of the drug using BSA standard curves^51–53^.

### In vitro studies

#### Cytotoxicity studies

The cytotoxicity of the samples was checked on the cancerous cell line, AGS by following the MTT (3-(4,5-dimethylthiazol-2-yl)-2,5-diphenyltetrazolium bromide) assay. For this study, AGS cells, having a density of 1 × 10^4^ (per well density), were seeded in 96 well plate and then allowed to become confluent up to 75–80%. After this, the cells were treated using three different concentrations (5, 7.5, and 10 µg/ml) of the FeCl_3_, FeSO_4_, LF, IONPs, and LF-IONPs. Following this treatment, cells were incubated in an incubator for 24 h and 37 °C. Post 24 h, MTT was added and again incubated for 3 h. Further, after incubation, the MTT, along with media, was removed from each and every well of the plate, and 200 µl of DMSO was added. Then, after 15 min, the absorbance was recorded at 570 nm.

To calculate the inhibition percentage, the following equation was used:

2 $$\% inhibition = [1 - (At/Ac) \times 100] \%$$

where At is the test substance absorbance and Ac is the control solvent absorbance.

Similarly, an MTT assay was performed on the cells treated with magnetic hyperthermia to check the effect of hyperthermia treatment on the cancer cells and evaluate the anti-cancer potential of synthesized nano-formulations. For this, the cells were grown in tissue culture plates (30 mm), followed by their treatment with FeCl_3_, FeSO_4_, LF, IONPs, and LF-IONPs and let be confluent. Thereafter, the cells were trypsinized and resuspended in HAM's media (100 µl) and, placed in a sample holder, and exposed to magnetic hyperthermia treatment for 10 min at a frequency of 242.4 kHz.

#### Scratch assay for anti-cancer activity

To confirm the anti-cancer activity of the synthesized formulations, a scratch assay was conducted. In this assay, cells were seeded in a 6-well plate and allowed to grow in an FBS-free medium (HAM's media) in an incubator at 37 °C and 5% CO_2_. Once the cells reached 80–90% confluency, a scratch was made using a pointed object such as a 10 µl tip. The cells were then washed with PBS to remove any debris, and different samples, including LF, iron oxide nanoparticles (IONPs), and LF-iron oxide nanospheres (LF-IONPs), were applied to the cells. The cells were then incubated for 6, 12, 24, 36, and 48 h, and images were taken at these mentioned intervals over a period of 48 h. To determine the percentage change in wound diameter for all the formulations, the distance of the wound was randomly measured at distinct positions for each scratch made in an individual well plate, and the mean of these independent readings was calculated. This experiment was repeated three times to record the data^54,55^.

## Results and discussions

### Structure and composition of LF-iron oxide nanoparticles

**Figure 1:**
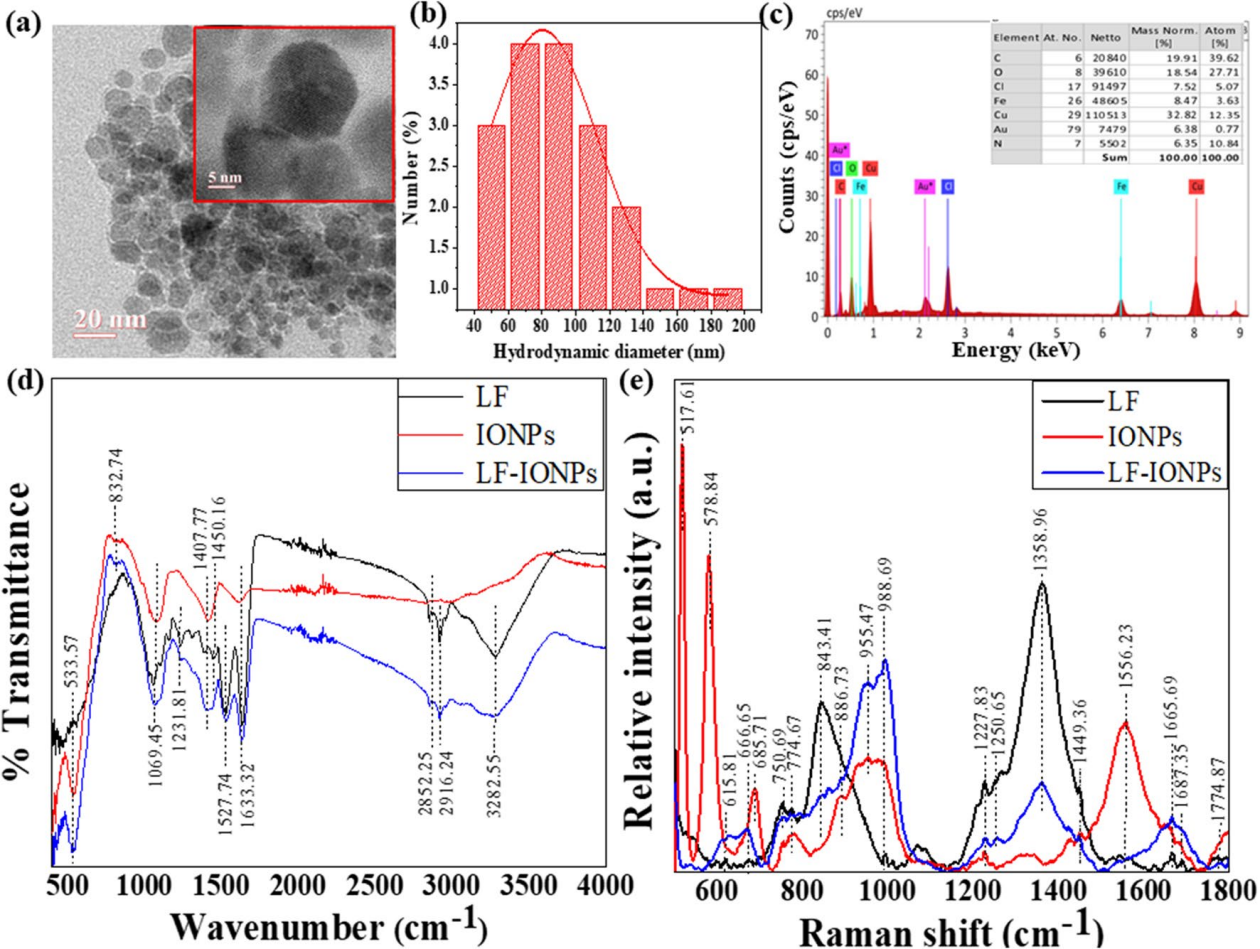
Characterization of LF-IONPs. (**a**) HR-TEM images on a scale of 20 nm with an inset showing 15 ± 2 nm size of LF-IONPs. (**b**) DLS showing the hydrodynamic size of the synthesized Lactoferrin-Iron oxide nanospheres (LF-IONPs) as 80.19 nm. (**c**) EDS showing that iron is present in LF-IONPs as 8.47%. (**d**) FTIR showing the interaction between protein LF and iron oxide nanoparticles. (**e**) Raman providing us with the structural fingerprint by which different molecules can be defined.

The formation of nanospheres having a spherical shape with an average size of 15 ± 2 nm was revealed by high-resolution transmission electron microscopic images (at a scale of 20 nm) as shown in Fig. 1a and inset of Fig. 1a (at a scale of 5 nm). Furthermore, the field emission scanning electron microscopic images (at a scale of 100 nm) depicted in Fig. S1a offer additional evidence that the iron oxide nanoparticles are embedded in the protein matrix. The hydrodynamic diameter of LF-IONPs was determined to be 80.19 nm, see Fig. 1b. Figure 1c displays the EDS (energy dispersive X-ray spectroscopy) spectrum, which clearly shows the presence of different elements, including C, N, O, Fe, Cl, Au and Cu. Notably, Fe was found to be evenly distributed, accounting for 8.47%.

### Study of interactions between LF and IONPs after nanoparticle formation using FTIR and Raman spectra

To investigate the interaction between protein and metal ions, FTIR analysis was conducted on LF samples, IONPs, and LF-IONPs. The results revealed several peaks in the IR spectra that provide insight into the molecular bonding present in the samples.

A peak was observed at 533.57 cm^−1^ in all three samples, indicating the presence of the Fe–O bond^56^. Another peak for Fe–N stretch was seen at 832.74 cm^−1^ in both IONPs and LF-IONPs^57^. Additionally, a peak at 1069.45 cm^−1^ was detected in all three samples, representing the C–N stretch^58^. Another peak at 1231.81 cm^−1^ was observed in LF and LF-IONPs but not in IONPs, indicating the bond Amide III^59^. The C=O stretching vibration was observed at 1407.77 cm^−1^ in all three samples^60^. Furthermore, the peaks at 1527.74 cm^−1^ and 1633.32 cm^−1^ representing the Amide II and Amide I bonds, respectively, were present in LF and LF-IONPs but not in IONPs^59^. A bond representing C–H stretching vibrations is present at 2852.25 cm^−1^ in lactoferrin and LF-IONPs but not seen in IONPs^61^. 2916.24 cm^−1^ represents a peak position for the bond O–H intermolecular interactions, which are observed in Lactoferrin and LF-IONPs but not in IONPs^61^. Also, Amine N–H stretching was observed at 3282.55 cm^−1^ in both lactoferrin and LF-IONPs but not in the case of IONPs^58^. This is shown in Fig. 1d and Table 1.

**Table 1:** The table gives the comparative values of the wavenumbers obtained from the FTIR (in the range of 400–4000 cm^−1^) and Raman spectra (500–1800 cm^−1^) respectively of lactoferrin, IONPs and LF-IONPs indicating changes in different functional groups present, thus coins the interaction among lactoferrin protein and iron oxide nanoparticles leading to the formation of LF-IONPs.

FTIR					Raman				
Functional groups	Lactoferrin (Lf)	IONPs	LF-IONPs	Reference	Functional groups	Lactoferrin (Lf)	IONPs	LF-IONPs	References
Fe–O	**533.57**	**533.57**	**533.57**	^56^	Fe–O Fe–O (T_2g_) Fe–O (A_1g_)	**–** **–** **666.65**	**517.61** **578.84** **685.71**	**–** **–** **666.65**	^62^
Fe–N stretch	**–**	**832.74**	**832.74**	^57^	Sulfur residues in cysteine	615.81	–	615.81	^64^
C–N stretch	1069.45	1069.45	1069.45	^58^	C–S stretch	750.69	–	750.69	^64^
Amide III	**1231.81** **1308.59**	–	**1231.81**	^59^	O–C–N (bend)	774.67	774.67	774.67	^59^
C=O stretching	1407.77	1407.77	1407.77	^60^	C–C stretching modes	843.41	886.73	843.41 886.73	^65^
Amide II	**1450.16** **1527.74**	–	**1527.74**	^59^	C–O–C	–	955.47	955.47	^59^
Amide I	**1633.32**	–	**1633.32**	^59^	Fe–O bond	**988.69**	**960.45**	**988.69**	^63^
C–H stretching vibrations	2852.25	–	2852.25	^61^	Amide III	**1227.83** **1250.65**	**–** **–**	**1227.83** **1250.65**	^66^
O–H Intramolecular stretching	2916.25	–	2916.24	^61^	C–H (bend)	1358.96	–	1358.96	^59^
Amine N–H stretching	3282.55	–	3282.55	^58^	Amide II	**1449.36**	**–**	**1449.36**	^59^
					Ferric hydroxide	**1556.23**	**1556.23**	**1556.23**	^63^
					Amide I	**1665.69**	**–**	**1665.69**	^59^
					C=O stretch	1774.87	–	1774.87	^59^

Significant values are in bold.

Raman analysis was performed for LF along with IONPs and LF-IONPs to identify different molecules based on their structural fingerprint. The peak observed at 517.61 cm^−1^ in IONPs represents Fe–O, while those at 578.84 cm^−1^ and 685.71 cm^−1^ indicate the bond Fe–O (T2g) and Fe–O (A1g), respectively, which are also present in LF and LF-Fe NPs but shifted to a lower wavenumber of 666.65 cm^−162^. Additionally, a peak at 960.45 cm^−1^ is observed in IONPs, while LF and LF-IONPs show a peak at 988.69 cm^−1^ for Fe–O^63^. The peak at 615.81 cm^−1^ signifies the presence of sulfur residues in cysteine, which is present in LF and LF-Fe NPs but absent in IONPs^64^. Similarly, the C–S stretch gives a peak at 750.69 cm^−1^ in LF and LF-IONPs but not in IONPs^64^. The peak at 774.67 cm^−1^ indicates the O–C–N bend, which is present in all three samples^59^. Another peak at 843.41 cm^−1^ indicates C–C stretching modes present in LF and LF-IONPs but shifted to a higher wavenumber of 886.73 cm^−1^ in IONPs^65^. The bond C–O–C is present in IONPs and LF-IONPs at a peak position of 955.47 cm^−1^ but absent in LF^59^. Amide III is present in LF and LF-IONPs at peak positions of 1227.83 cm^−1^ and 1250.65 cm^−1^, respectively^66^. A peak at 1358.96 cm^−1^ indicates C–H (bend), which is observed in both LF and L-IONPs but not in IONPs^59^. The peak at 1449.36 cm^−1^ signifies the presence of the Amide II bond in LF and LF-IONPs^59^. A ferric hydroxide peak is seen at 1556.23 cm^−1^, which is present in all three samples^63^. Amide I bond at 1665.69 cm^−1^ is present in both LF and LF-IONPs but absent in IONPs^59^. Finally, the C=O stretch was observed in LF and LF-IONPs at 1774.87 cm^−1^, but no peak was observed in IONPs at this wavenumber^59^. These results are presented in Fig. 1e and Table 1.

Overall, the results from FTIR and Raman spectroscopy indicate differences in the chemical bonding between LF and its interactions with metal ions, providing insights into the interaction between protein and metal ions. The key findings from the FTIR and Raman spectroscopy analyses indicate distinct molecular bonding interactions between LF and metal ions, as evidenced by the presence of Fe–O, C–N, Amide III, Amide II, and Amide I bond in LF and LF-IONPs, but not in IONPs. These results provide valuable insights into the specific nature of the protein-metal ion interactions.

### In silico studies to determine the interactions between Fe^2+^ and Fe^3+^ and LF protein

We utilized the MIB (metal ion binding) software, an online docking server, to predict the binding sites of LF with Fe^2+^ and Fe^3+^. The interaction between metal ions and amino acid residues in the protein depends on its structure and sequence. We extracted human LF protein from PDB (PDB ID: 1B0L) and docked it with both metal ions using MIB. Each binding residue in LF protein was assigned a binding score, and residues with scores higher than the threshold were considered. The binding sites for Fe^2+^ and Fe^3+^ were determined, and the distance between the metal ions and their binding site on the LF chain was calculated using Maestro.

**Figure 2:**
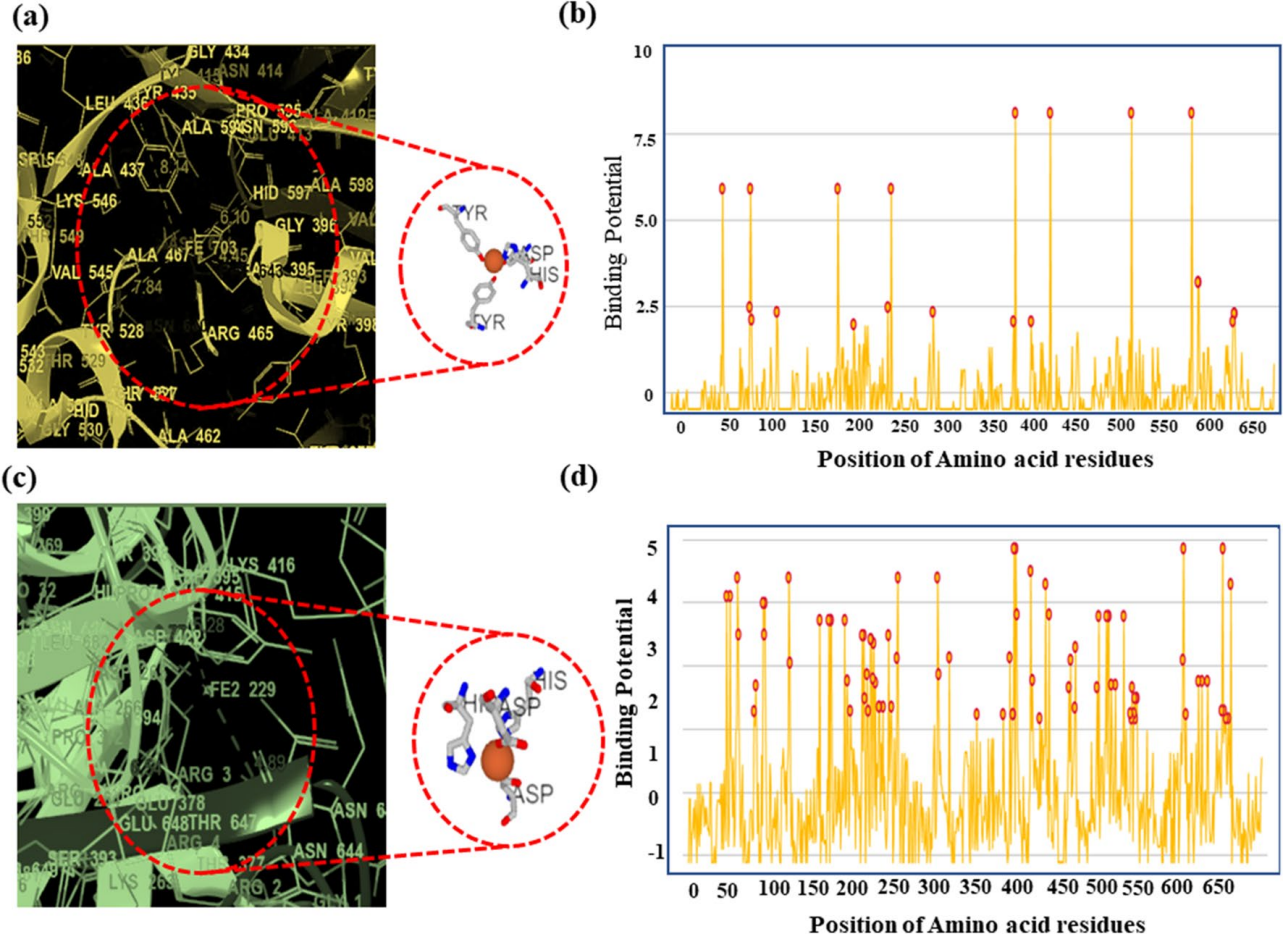
Metal ion binding residues showing binding of Fe^3+^ and Fe^2+^ with amino acids on the chain of the lactoferrin protein. (**a**) Binding of Fe^3+^ with amino acids 435Y, 597H, 395D, 528Y, and their distance was measured to be 8.14 Å, 6.10 Å, 4.45 Å, 7.84 Å respectively. (**b**) Binding of Fe^2+^ with amino acids 393S, 395D, 597H, 644N with a distance of 6.4 Å, 5.28 Å, 3.77 Å, 4.89 Å, respectively.

For Fe^3+^, it was observed that there were three binding sites at specific amino acid residues: (1) 435Y, 597H, 395D, and 528Y, and their distance was measured to be 8.14 Å, 6.10 Å, 4.45 Å, 7.84 Å respectively. (2) 60D, 92Y, 192Y, 253H, and they were at a distance of 4.73 Å, 8.18 Å, 7.89 Å, and 6.14 Å respectively and (3) 604D, and 605 K with a distance of about 4.65 Å, and 4.65 Å, respectively, as shown in Fig. 2a and Fig. S2. Further, the binding potential graph of Fe^3+^ with different amino acid residues is illustrated in Fig. 2b.

For Fe^2+^, there were seven binding sites involving specific amino acids for each site with varying distances from metal ions: (1) 393S, 395D, 597H, 644N with a distance of 6.4 Å, 5.28 Å, 3.77 Å, 4.89 Å respectively. (2) 393S, 413E, 597H, and 644N with distances were found 6.59 Å, 4.99 Å, 3.06 Å, and 5.23 Å, respectively. (3) 60D, 122 T, 253H, 301 K, and their distance were 4.39 Å, 7.05 Å, 3.84 Å, and 7.73 Å, respectively. (4) 60D, 122 T, 253H, 301 K, which showed a distance of 4.45 Å, 7.92 Å, 3.90 Å, and 6.50 Å, respectively, between the metal ion and the amino acids. (5) 431P, 654H with a distance of 4.81 Å, 3.45 Å respectively. (6) 60D, 122 T, 253H, 301 K and showed a distance of about 4.85 Å, 7.39 Å, 4.41 Å, and 5.94 Å, respectively, and (7) 47Q, 51E with a distance of 8.35 Å, and 6.77 Å, respectively. This is shown in Fig. 2c and Fig. S3. Moreover, for Fe^2+^, the binding potential is shown in Fig. 2d.

**Figure 3:**
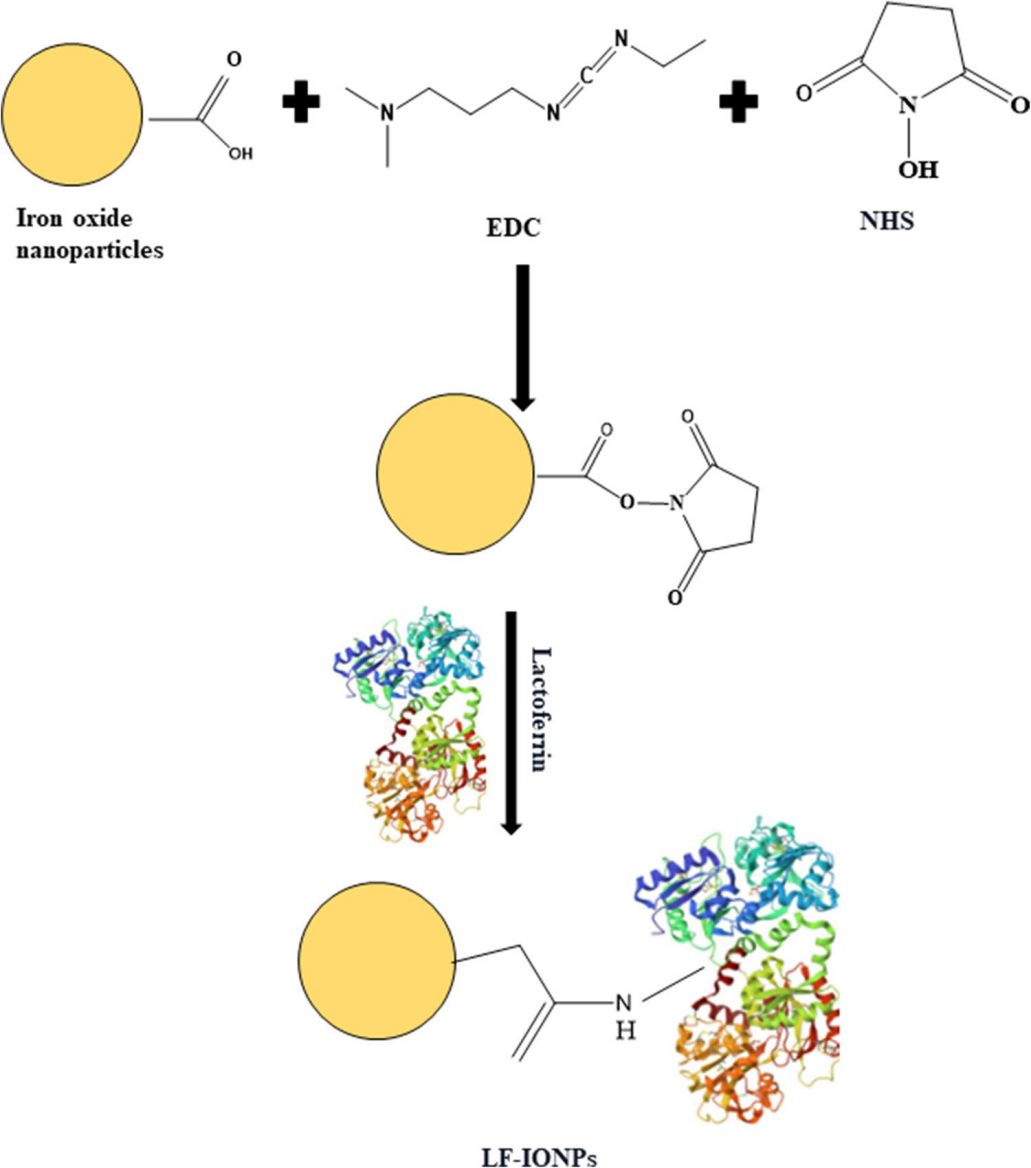
The figure shows the schematic representation of the synthesis procedure. It depicts the schematic representation for the conjugation of lactoferrin protein with iron oxide nanoparticles, which is achieved through EDC/ NHS coupling reaction.

A schematic representation of the conjugation of lactoferrin to IONPs by employing EDC/NHS coupling reaction to synthesize LF-IONPs is illustrated in Fig. 3.

### XRD analysis

X-ray diffraction was conducted to assess the crystallinity of the materials. Fig. S1b shows the XRD diffraction patterns of iron oxide nanoparticles (IONPs), lactoferrin protein (LF), and lactoferrin-iron oxide nanoparticles (LF-IONPs). The diffraction peaks observed in XRD patterns of IONPs appearing at 2Ɵ = 30°, 35°, 43°, 52°, 57°, 62° correspond to crystal planes (220), (311), (400), (422), (511), and (440). These peaks match well with the peaks shown in 00-003-0863 ICDD number for Fe_3_O_4._ The diffraction patterns for iron oxide in lactoferrin-iron oxide nanoshperes (LF-IONPs) were observed at the same position as those in iron oxide particles, suggesting that the addition of lactoferrin to the iron oxide nanoparticles does not significantly alter their crystalline structure. On the other hand, in the XRD pattern of lactoferrin protein alone, a peak with a broad hump was observed in the range of 23°–25°, which indicates the presence of an amorphous material, as it lacks the distinct and sharp peak associated with crystalline structure. The crystallite size of IONPs and LF-IONPs was determined by the Scherrer formula (Eq. 1) and is reported in Table 2.

**Table 2:** Saturation magnetization (M_S_), remanence (M_r_), coercivity (H_C_) and crystallite size of bare IONPs and lactoferrin coated iron oxide nanoparticles (LF-IONPs).

Parameters	Samples	
	IONPs	LF-IONPs
M_S_ (emu/g)	50.88	50.04
M_r_ (emu/g)	0.03	0.04
H_C_ (Oe)	0.26	0.68
Crystallite size (nm)	15.55	17.77

### Hyperthermia study

**Figure 4:**
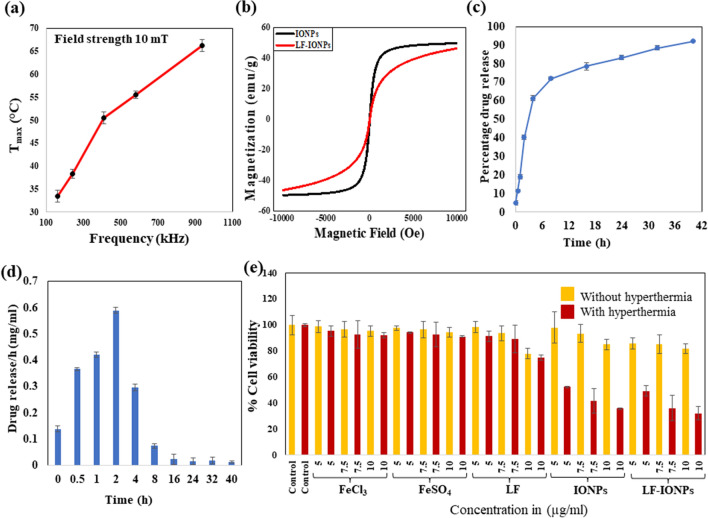
Determination of hyperthermia responsiveness by LF-IONPs. (**a**) The figure illustrates the heating capacity of LF-Iron oxide nanospheres at different frequencies with a constant magnetic field of 10 mT. It shows the temperature rise, i.e., 33.26 °C, 38.34 °C, 50.56 °C, 55.54 °C, and 66.26 °C at different frequencies; 161.9 kHz, 242.4 kHz, 411.1 kHz, 580.2 kHz, and 935.3 kHz, respectively. (**b**) M-H loops of IONPs and LF-IONPs measured at room temperature. (**c**) The plot displays the release kinetic studies conducted to determine the percentage of drug release from LF-IONPs at pH 3. (**d**) The graph represents the drug release per h in mg/ml at pH 3. (**e**) The MTT assay determined the cytotoxic activity of AGS cells in the absence and presence of hyperthermia. The data show the treatment of AGS cells with control, including salt solutions FeCl_3_ and FeSO_4_, LF, IONPs, and LF-IONPs at concentrations 5 µg/ml, 7.5 µg/ml, and 10 µg/ml for each sample, both in the absence and presence of hyperthermia treatment. The data were plotted as the mean of three independent experiments.

To understand the magnetic hyperthermia heating effects of the nanoparticles, the temperature vs time profiles were recorded under varying frequencies while keeping the field strength and time constant. At each frequency, the sample was subjected to the hyperthermia treatment for 10 min as in this period of time, hyperthermia temperature is achieved, and beyond this time, overheating takes place. While conducting the experiment, the initial temperature of the machine was 30 °C. Further, it was found that as we increase the frequency from 161.9 to 242.4 kHz, 411.1 kHz, 580.2 to 935.3 kHz, there is a linear increase in temperature generated from 33.26 to 38.34 °C, 50.56 °C, 55.54 to 66.26 °C, respectively, as illustrated in Fig. 4a which shows a temperature (T_max_) Vs frequency (kHz) plot*.* This temperature rise indicates that the nanoparticles have potential for use in magnetic hyperthermia treatment of cancerous tissues. SAR values were also calculated at above mentioned frequencies which is given in Table S1.

### Magnetic properties

Magnetic properties of bare and lactoferrin coated iron oxide nanoparticles were determined magnetization measurement. M–H measurements were performed at room temperature on Lakeshore 7404 model in the field range of ± 10 kOe. M–H loops of IONPs and LF-IONPs are presented in Fig. 4b. From these hysteresis loops, saturation magnetization (M_S_), Coercivity (H_C_) and remanence (M_r_) are determined, which are reported in Table 2. From Table 2, it can be seen that both remanence and coercivity of IONPs and LF-IONPs are nearly zero indicating that the synthesized nanoparticles are superparamagnetic. This indicates that at room temperature thermal energy can overcome the magnetic anisotropy energy and keeps them randomly oriented in the absence of external magnetic field. Further, LF-IONPs also possess high saturation magnetization making them a suitable candidate for the magnetic hyperthermia applications.

### Drug loading and release kinetics

The encapsulation efficiency of LF protein over IONPs was found to be 93 ± 0.014%. The drug release was monitored at pH 7.4 and pH 3. No drug release was observed at the physiological pH of 7.4. However, when we perform the studies at gastric pH, drug was observed to have a burst release for the first 8 h followed by a sustained release for the next ~ 30 h. At the end of 40 h, 92.18 ± 0.003% of the drug was released, highlighting the efficiency of LF-IONPs as a drug delivery system (Fig. 4c,d) and shown in Fig. S4.

### In-vitro cell line studies in the presence and absence of hyperthermia

To evaluate the cytotoxicity of LF-IONPs on the cancer cell line AGS, we performed an MTT assay under physiological conditions. The cytotoxicity was calculated for FeCl_3_ and FeSO_4_ salt solutions, LF protein, IONPs, and LF-IONPs at three concentrations: 5 µg/ml, 7.5 µg/ml, and 10 µg/ml. Three independent sets were prepared for each concentration, along with control. The assay was performed both with and without hyperthermia treatment to determine the efficacy of the treatment.

In the MTT assay without hyperthermia treatment, cells treated with FeCl_3_ salt solution showed cell viability of 98.67 ± 4.5% at 5 µg/ml, 96.65 ± 5.8% at 7.5 µg/ml, and 95.38 ± 4% at 10 µg/ml. Similarly, FeSO_4_-treated cells showed cell viability as 97.61 ± 1.62% at 5 µg/ml, 96.44 ± 6.15% at 7.5 µg/ml, and 94.32 ± 3.74% at 10 µg/ml. Cells treated with LF showed cell viability of 98.35 ± 1.2% at 5 µg/ml, 93.58 ± 5.8% at 7.5 µg/ml, and 77.87 ± 4.5% at 10 µg/ml. For cells treated with IONPs, the cell viability was 98.06 ± 11.80%at 5 µg/ml, 93.48 ± 7.03%at 7.5 µg/ml, and 85.03 ± 3.95%at 10 µg/ml. Finally, cells treated with LF-IONPs showed cell viabilityof 85.56 ± 4.25%at 5 µg/ml, 85.21 ± 7.06%at 7.5 µg/ml, and 81.69 ± 3.76%at 10 µg/ml concentration.

After the incubation period, the cells were subjected to hyperthermia treatment (at a frequency of 242.4 kHz for 10 min), and an MTT assay was performed to determine the effect of synthesized formulations on cell viability. The treated cells showed a significant increase in cytotoxicity compared to the control group. For FeCl_3_ salt solution-treated cells, the cell viability was 95.38 ± 3.9% at 5 µg/ml, 92.62 ± 10.4% at 7.5 µg/ml, and 91.88 ± 2% at 10 µg/ml. When cells were treated with FeSO_4_ salt solution, the cell viability was 94.11 ± 0.77% at 5 µg/ml, 92.73 ± 9.54% at 7.5 µg/ml, and 91.14 ± 0.49% at 10 µg/ml. For LF-treated cells, the cell viability levels were 61.53 ± 3.9%, 60.26 ± 10.4%, and 40.95 ± 2% for the concentrations 5 µg/ml, 7.5 µg/ml, and 10 µg/ml, respectively. On the other hand, cells treated with IONPs showed cell viability levels of 52.36 ± 0.77% for 5 µg/ml, 41.53 ± 9.54% at 7.5 µg/ml, and 35.70 ± 0.49% at 10 µg/ml. Lastly, LF-IONPs resulted in cell viability levels of 49.33 ± 4%, 36.18 ± 9.7%, and 32.25 ± 5.2% for 5 µg/ml, 7.5 µg/ml, and 10 µg/ml, respectively. These results provide substantial evidence of the cytotoxic nature of the synthesized nanospheres against cancerous cells, which can be further explored for anti-cancerous activity. The data is shown in Fig. 4e. Table shows the p values calculated for % variation in cell viability after treatment with 5, 7.5 and 10 µg/ml of IONPs and LF-IONPs with and without hyperthermia. The statistical significance of data is considered when the p < 0.05 and the comparative data are presented in Table S2.

### Scratch assay for anti-cancer activity

**Figure 5:**
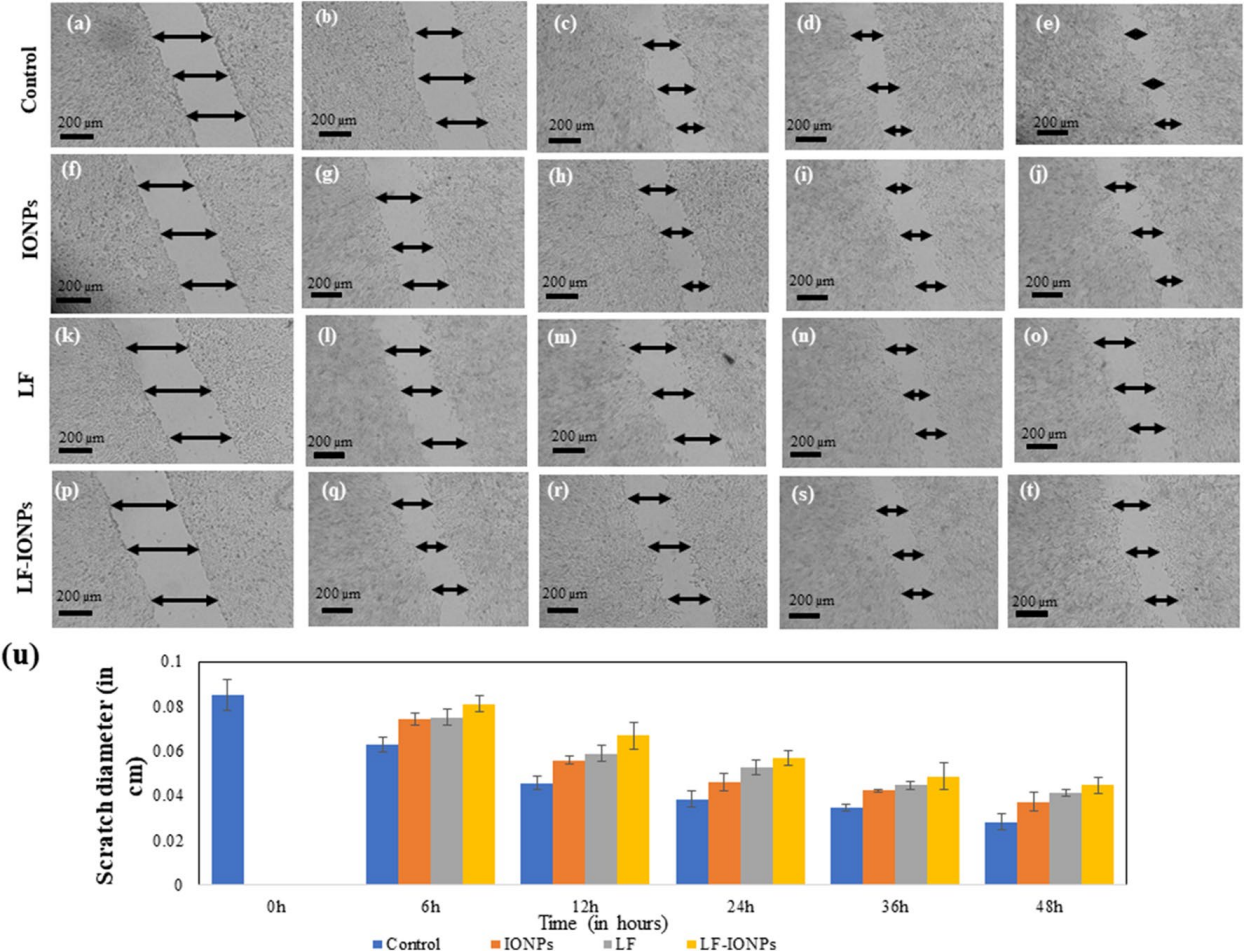
The figure shows the anti-cancer effect of nanoparticles using AGS cell line via scratch assay. (**a**) 6 h, (**b**) 12 h, (**c**) 24 h, (**d**) 36 h, (**e**) 58 h are control cells, IONPs treated cells after (**f**) 6 h, (**g**) 12 h, (**h**) 24 h, (**i**) 36 h, (**j**) 48 h at a fixed concentration of 10 µg/ml, LF treated cells after (**k**) 6 h, (**l**) 12 h, (**m**) 24 h, (**n**) 36 h, (**o**) 48 h with a concentration of 10 µg/ml, Cells treated with LF-IONPs after (**p**) 6 h, (**q**) 12 h, (**r**) 24 h, (**s**) 36 h, (**t**) 48 h with 10 µg/ml concentration, (**u**) Plot showing the comparison of the change in scratch diameter post-treatment with LF, IONPs, and LF-IONPs including control after 6 h, 12 h, 24 h, 36 h, and 48 h.

In order to assess the impact of LF-IONPs on the migration ability of AGS cells, a scratch assay was employed in which the cells were exposed to a fixed concentration (10 µg/ml) of LF-IONPs, LF, and Iron oxide nanoparticles (IONPs). To determine the percentage change in the scratch diameter, the diameter was measured at three distinct positions for each scratch, and the mean diameter of individual readings of each scratch was calculated. Control cells exhibited gap percentages of 26.19 ± 0.35%, 46.42 ± 0.30%, 54.76 ± 0.35%, and 66.66 ± 0.35% after 6, 12, 24, 36, and 48 h, respectively. In contrast, the cells treated with IONPs showed gap percentages of 12.85 ± 0.26%, 34.52 ± 0.17%, 46.19 ± 0.39%, 50.47 ± 0.07%, and 56.19 ± 0.44% after the same time intervals. Furthermore, LF-treated cells displayed even greater inhibition at 6, 12, 24, 36, and 48 h with percentages of 11.90 ± 0.35%, 30.95 ± 0.35%, 38.09 ± 0.35%, 47.61 ± 0.17%, and 51.42 ± 0.16%, respectively. The LF-IONPs treatment showed the most inhibition, with percentages of 4.76 ± 0.35%, 21.42 ± 0.60%, 33.33 ± 0.35%, 42.85 ± 0.60%, and 47.61 ± 0.35% after 6, 12, 24, 36, and 48 h, respectively. The results indicated that LF-IONPs treatment significantly inhibited cell migration compared to control cells, which almost filled the gap within 48 h. These findings further support the nanoparticles' anti-cancerous properties, as demonstrated in Fig. 5. To determine the statistical significance of the data, p values were calculated for the scratch assay after treatment, and the comparative data are presented in Table S3.

## Conclusion

To investigate the potential of nanoparticles as an anti-cancer agent, we used lactoferrin protein as a capping agent and iron oxide nanoparticles. Both of these components have demonstrated anti-cancer activities. Lactoferrin interacts with high levels of proteoglycan, glycosaminoglycan, and sialic acid found in cancer cells and activates the signaling pathways to exert cytotoxic effects on gastric cancer cells. Iron oxide nanoparticles, which we synthesized by using the co-precipitation method, were linked with lactoferrin protein by EDC-NHS, which activates the carboxyl group on the surface of iron oxide particles, facilitating their conjugation with the protein. The synthesized particles exhibited a desirable HR-TEM size of about 15 ± 2 nm and demonstrated notable heating capacity when exposed to the external magnetic field.

By successfully conjugating lactoferrin with iron oxide nanoparticles, we achieved targeted delivery to gastric cancer cells, as lactoferrin is known to interact with proteoglycan, glycosaminoglycan, and sialic acid, which are present on the surface of cancer cells. Furthermore, lactoferrin and iron oxide nanoparticles showed potent synergistic anti-cancer activity against cancer cell lines at a very low concentration. These particles were easily internalized in the AGS cell line, and the synthesized LF-IONPs (lactoferrin conjugated iron oxide nanospheres) showed a remarkable increase in anti-cancer properties as compared to individual components. This significantly enhanced efficacy, combined with a great degree of selectivity, can be attributed to the conjugation of LF and IONPs.

The remarkable superparamagnetic behavior, substantial magnetization capacity, and excellent heating capacity of these nanospheres hold great promise for their use in numerous areas of biomedical research applications. We strongly believe that these synthesized particles have great potential to target different types of cancer cell lines effectively. It is our hope that the findings of this paper will inspire further research for the future use of these anti-cancer nanoparticles.

### Supplementary Information

Supplementary Information.
