# Supplementary material for: Therapeutic potential of lactoferrin-coated iron oxide nanospheres for targeted hyperthermia in gastric cancer
Source: Sci Rep. 2023 Oct 19;13:17875. doi: 10.1038/s41598-023-43725-3 (PMC10587155; doi:10.1038/s41598-023-43725-3)
Supplement: Supplementary file 1 — Supplementary Information. [file 41598_2023_43725_MOESM1_ESM.docx]

**Therapeutic Potential of Lactoferrin-Coated Iron Oxide Nanospheres for Targeted Hyperthermia in Gastric Cancer**

Komal Attri^1,4^, Bhupendra Chudasama^2,4^*, Roop L. Mahajan^3,4*^, Diptiman Choudhury^1,4^*

^1^School of Chemistry and Biochemistry, Thapar Institute of Engineering and Technology, Patiala-147004, Punjab, India.

^2^School of Physics and Material Sciences, Thapar Institute of Engineering and Technology, Patiala-147004, Punjab, India

^3^Department of Mechanical Engineering, Virginia Tech, Blacksburg, VA 24061, United States; Institute for Critical Technology and Applied Science, Virginia Tech, Blacksburg, VA 24061, United States.

^4^TIET-VT Centre of Excellence for Emerging Materials, Thapar Institute of Engineering and Technology, Patiala-147004 Punjab, India.

*****Corresponding E-mail: [diptiman@thapar.edu](mailto:diptiman@thapar.edu), [mahajanr@vt.edu](mailto:mahajanr@vt.edu), bnchudasama@thapar.edu

*Corresponding Phone: +91-8196949843 / +1-5402312597 / +91-9781966136


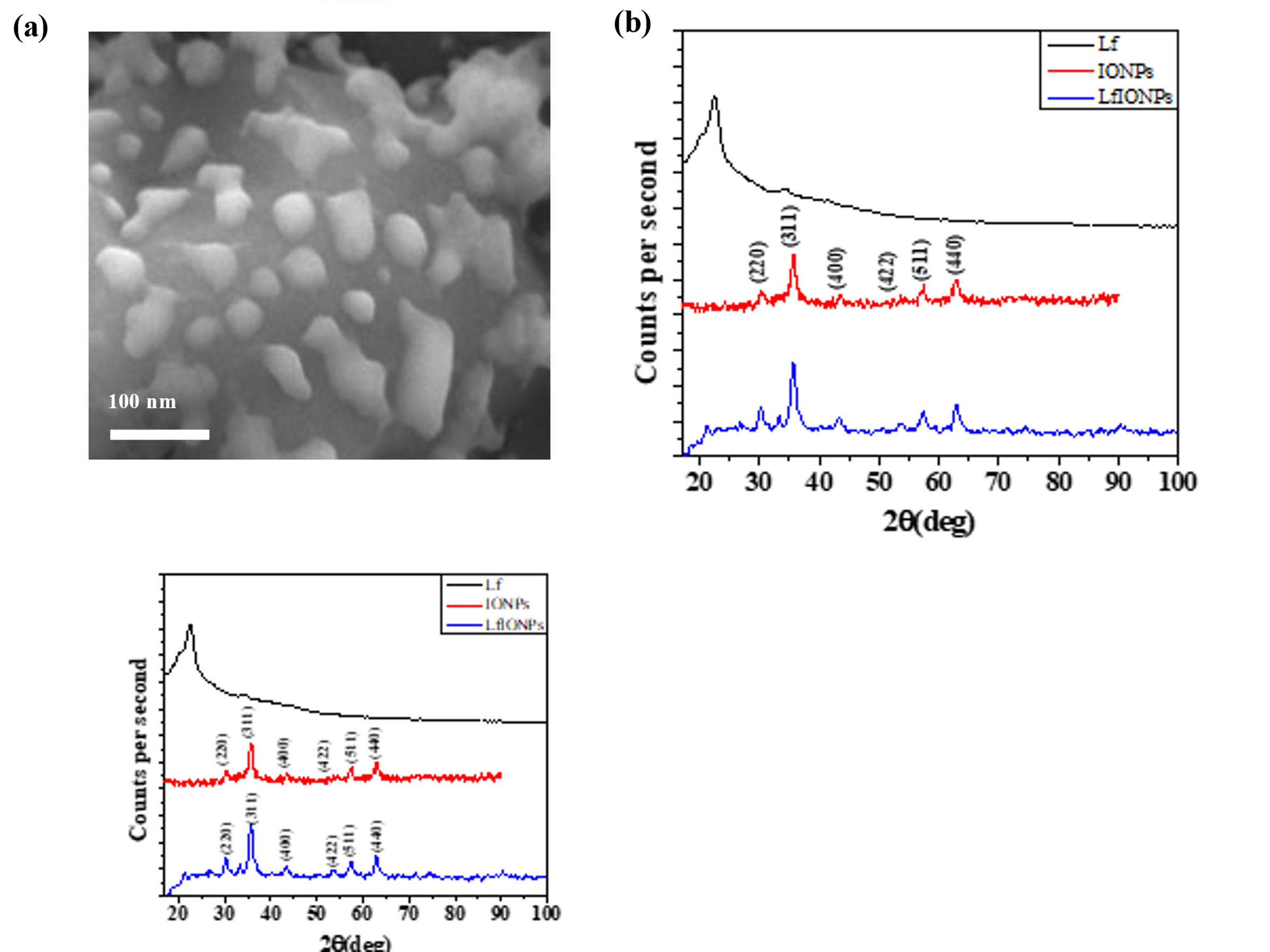


**Figure S1**: (a) Fe-SEM micrograph of LF-IONPs on a scale of 100 nm (b) XRD graph shows the X-Ray diffraction pattern which gives deep knowledge about the chemical composition, crystallographic structure and physical properties of a materials including FeCl_3_, FeSO_4_, IONPs, LF and LF-IONPs when subjected to the Cu K α irradiation.


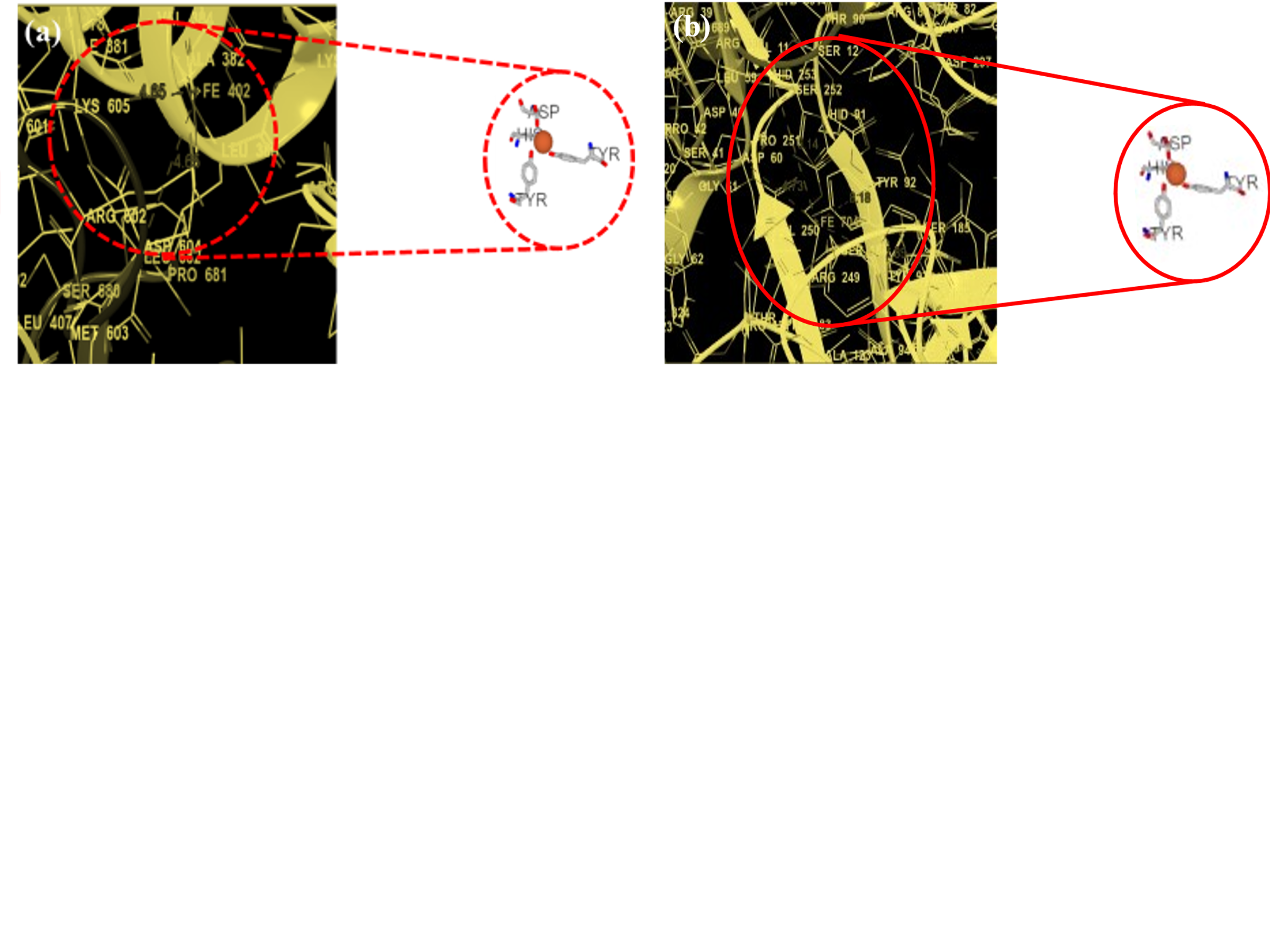


**Figure S2:** Metal ion binding residues showing binding of Fe^+3^ with amino acids on the chain of the lactoferrin protein (a) Binding of Fe^+3^ with amino acids 60D, 92Y, 192Y, 253H and they were at a distance of 4.73 Å, 8.18 Å, 7.89 Å, 6.14 Å respectively (b) Binding of Fe^+3^ with amino acids 604D, 605K with a distance of about 4.65 Å, 4.65 Å respectively.


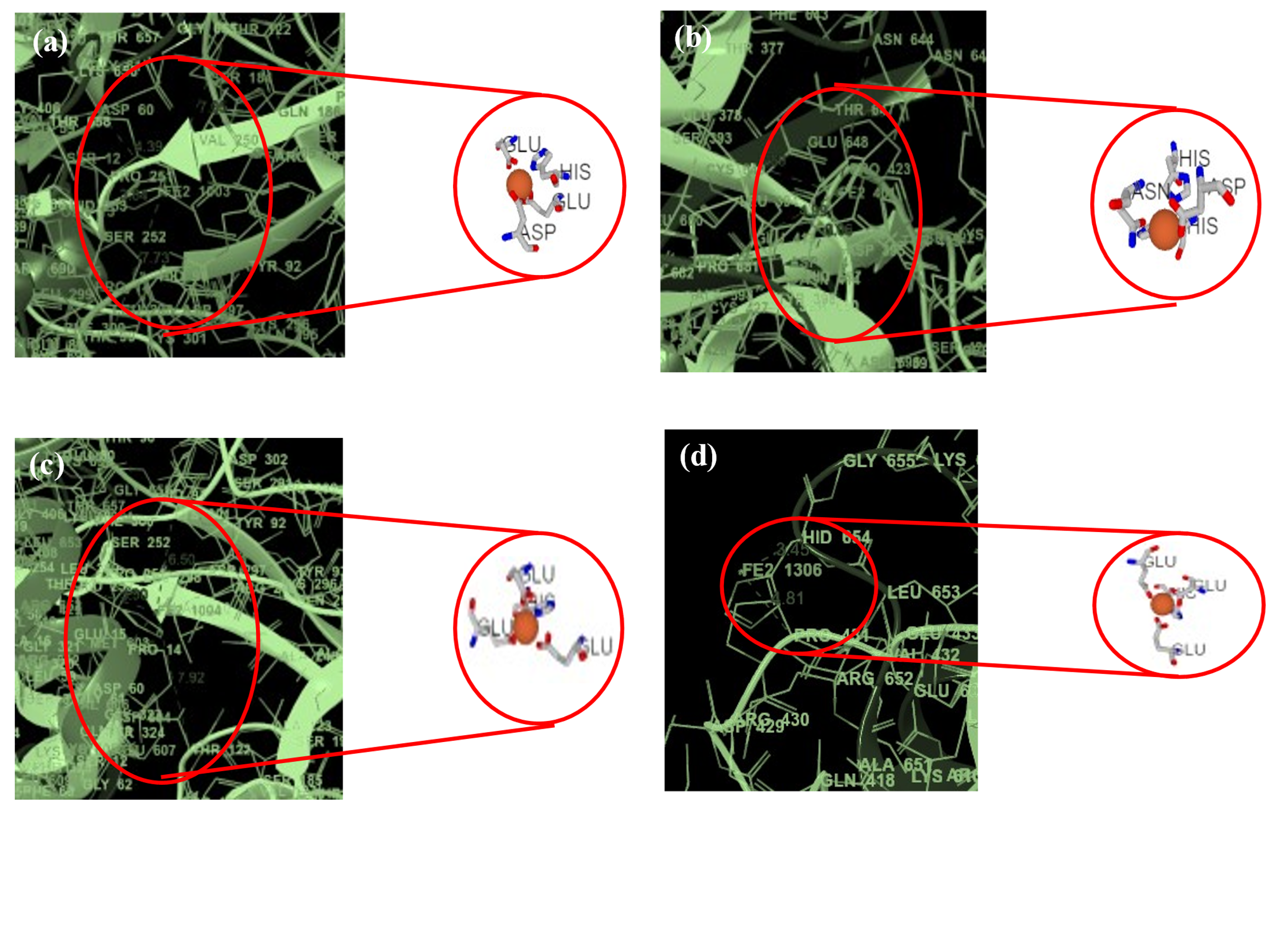


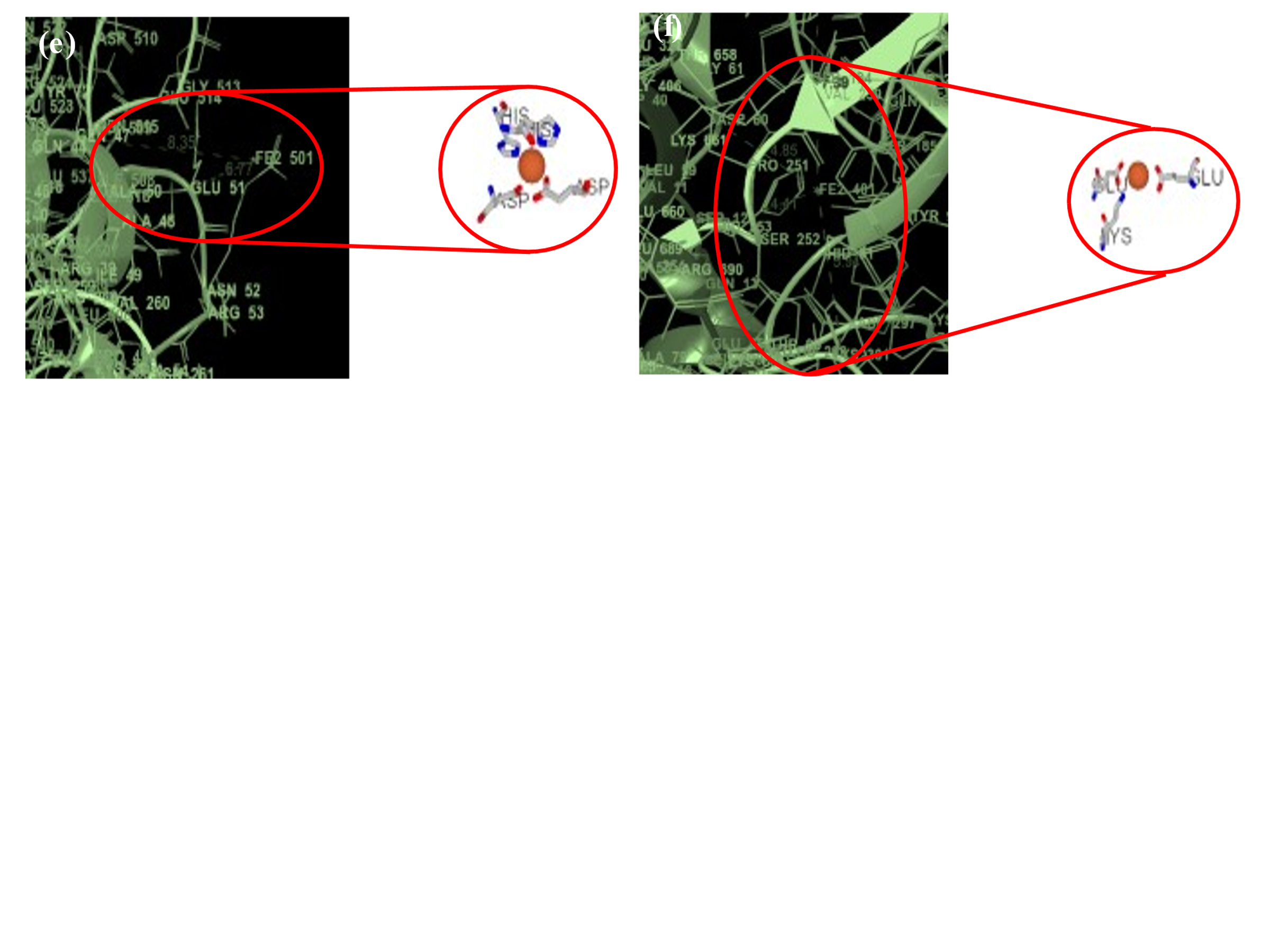


**Figure S3** Metal ion binding residues showing binding of Fe^+2^ with amino acids on the chain of the lactoferrin protein (a) Binding of Fe^+2^ with amino acids 393S, 413E, 597H, 644N, and distance was found out to be 6.59 Å, 4.99 Å, 3.06 Å, 5.23 Å respectively (b) Binding of Fe^+2^ with amino acids 60D, 122T, 253H, 301K and their distance were as such 4.39 Å, 7.05 Å, 3.84 Å,7.73 Å respectively (c) Binding of Fe^+2^ with amino acids 60D, 122T, 253H, 301K which showed a distance of 4.45 Å, 7.92 Å, 3.90 Å, 6.50 Å respectively (d) Binding of Fe^+2^ with amino acids 431P, 654H with a distance of 4.81 Å, 3.45 Å respectively (e) Binding of Fe^+2^ with amino acids 60D, 122T, 253H, 301K and showed a distance of about 4.85 Å, 7.39 Å, 4.41 Å, 5.94 Å respectively (f) 47Q, 51E with a distance of 8.35 Å, 6.77 Å respectively.

**Figure S4** The plot displays the release kinetic studies conducted to determine the percentage of drug release from LF-IONPs at pH 7.4 and pH 3.

**Table S1.** The table shows the SAR (Specific Absorption Rate) and ILP (Intrinsic Loss Power) values for the LF-IONPs at different frequencies.

| **Frequency (kHz)** | **SAR (Specific Absorption Rate)** | **ILP (Intrinsic Loss Power)** |
| --- | --- | --- |
| 161.9 kHz | 3.49 ± 0.449 W/g | 0.34 ± 0.044 nHm^2^/k |
| 242.4 kHz | 9.71 ± 0.722 W/g | 0.63 ± 0.047 nHm^2^/k |
| 411.1 kHz | 37.46 ± 3.397 W/g | 1.43 ± 0.130 nHm^2^/k |
| 580.2 kHz | 49.86 ± 3.477 W/g | 1.35 ± 0.094 nHm^2^/k |
| 935.3 kHz | 75.95 ± 6.279 W/g | 1.28 ± 0.106 nHm^2^/k |

**Table S2.** The table shows the p values calculated for % variation in cell viability after treatment with 5, 7.5, and 10 µg/ml of IONPs and LF-IONPs with and without hyperthermia. The statistical significance of data is considered when the p < 0.05

| **P value for checking the statistical significance of data** | | |
| --- | --- | --- |
|  | **IONPs** | **LF-IONPs** |
| 5µg/ml | 0.0079 | 0.0136 |
| 7.5 µg/ml | 0.0231 | 0.0350 |
| 10 µg/ml | 0.0021 | 0.0085 |

**Table S3.** The table shows the p values calculated for % variation in scratch diameter after treatment with 10 µg/ml of FeCl_3_, FeSO_4_, lactoferrin, IONPs, and LF-IONPs. The statistical significance of data is considered when the p < 0.05.

| **P value for checking the statistical significance of data** | | | |
| --- | --- | --- | --- |
| **Time** | **LF** | **IONPs** | **LF-IONPs** |
| **6h** | 0.013236 | 0.011081 | 0.003126 |
| **12h** | 0.007966 | 0.00749 | 0.005609 |
| **24h** | 0.007763 | 0.073926 | 0.003126 |
| **36h** | 0.001935 | 0.001685 | 0.004992 |
| **48h** | 0.003772 | 0.051658 | 0.004813 |
